# IView: introgression library visualization and query tool

**DOI:** 10.1186/1471-2105-11-S6-S28

**Published:** 2010-10-07

**Authors:** Christopher A Bottoms, Sherry Flint-Garcia, Michael D McMullen

**Affiliations:** 1 Division of Plant Sciences, University of Missouri, Columbia, MO 65211, USA; 2 Plant Genetics Research Unit, USDA-Agricultural Research Service, Columbia, MO 65211, USA

## Abstract

**Background:**

An introgression library is a family of near-isogenic lines in a common genetic background, each of which carries one or more genomic regions contributed by a donor genome. Near-isogenic lines are powerful genetic resources for the analysis of phenotypic variation and are important for map-base cloning genes underlying mutations and traits. With many thousands of distinct genotypes, querying introgression libraries for lines of interest is an issue.

**Results:**

We have created IView, a tool to graphically display and query near-isogenic line libraries for specific introgressions. This tool incorporates a web interface for displaying the location and extent of introgressions. Each genetic marker is associated with a position on a reference map. Users can search for introgressions using marker names, or chromosome number and map positions. This search results in a display of lines carrying an introgression at the specified position. Upon selecting one of the lines, color-coded introgressions on all chromosomes of the line are displayed graphically.

The source code for IView can be downloaded from http://xrl.us/iview.

**Conclusions:**

IView will be useful for those wanting to make introgression data from their stock of germplasm searchable.

## Background

Near isogenic lines (NILs) are lines derived from a particular parental line (i.e. the recurrent parent) that carry genomic regions of another line (i.e. the donor parent). An introgression library is a family of NILs with common recurrent and donor parents. The NILs are created by crossing the donor and recurrent parents, and backcrossing the F1 and subsequent generations with the recurrent parent, thereby reducing the amount of donor genome in each generation. The resulting offspring are then self-pollinated several generations to produce the NILs. The genomes of the NILs are primarily that of the recurrent parent, with one or more regions of the genome originating from the donor parent genome (i.e. introgressions). Each NIL in a family will contain different combinations of introgressions.

Introgression libraries are useful for testing the phenotypic effects of donor introgressions and as the starting material for map-based cloning populations.  Since NILs are genetically similar to the recurrent parent except for the genomic region of interest, they can be used to test the phenotypic effects of the donor region. Fine mapping studies are initiated by choosing a NIL with the smallest introgression surrounding a given region of the genome. Being able to quickly query an introgression library for introgressions of interest facilitates their use in genetic studies.

## Implementation

IView is a Perl-based web application. Prior to setting up the web interface, users provide input files that allow determining introgressions. Data are stored in a MySQL database. Template Toolkit templates are used for the web pages. IView is currently configured to run using the "Plack" server that is automatically installed along with the source code. IView has been tested and works well on 64-bit Debian Lenny Linux. 

## Installation

### Installation on Debian Linux

A Debian Linux-specific installation *bash* script can be downloaded from http://xrl.us/iviewdebbsh.  This script can be used to install everything that is needed to run IView. If you do not have super-user privileges, then contact the system administrator to ensure that the following packages are installed: *mysql-server-5.0*, *make*, *gcc*, and *libgd2-xpm-dev*. Otherwise, the installation script will ask for the “sudo“ password so that it can install these automatically. 

To use the installation script, first open a terminal window. To do so, click on "Applications", then "Accessories", and then "Terminal". Please remember that Linux is case sensitive. Type the following commands, pressing <ENTER> after each one (commands are in the font Courier New). 

wget http://xrl.us/iviewdebbsh

chmod u+x iview-deb.bsh

./iview-deb.bsh

The script will install the MySQL relational database management system, if it is not already installed. During installation of MySQL, a window will open that will request that you create a password for the MySQL root user. Note that this is distinct from the system root. Enter the password that you wish to create and then press <ENTER>. As is usual when creating passwords, you will then be asked to re-enter the new password. After entering it again, press <ENTER>.  Please keep track of this password, as it will be needed later. 

For all other questions that occur during installation, the user can choose to accept the defaults by simply pressing <ENTER>.

This command will start installation of multiple programs and takes about twenty minutes.

### Setting up the program with the sample data set

These instructions should work equally well for users wanting to view their own introgression data. These instructions pick up at the end of the installation instructions. As part of the installation process, the directory “IView” should have been created. To change to this directory and run the sample, type the following two commands, pressing <ENTER> after each one:

cd IView

perl setup_site.pl sample.cfg

This last command processes a sample dataset. You will be asked for the MySQL root password (that you created earlier) so that a dummy user can be created for accessing the sample database.

To start the web server, type the following commands, pressing <ENTER> after each one:

cd cgi-bin

plackup sample.psgi

Now, please minimize the terminal window, since it is now tied up by output from the web server. When you need to stop the web server, click in this terminal window and press the <Ctrl> key at the same time as the <C> key. 

Now open another terminal and type the following and then press <ENTER>:

firefox http://localhost:5000/sample

**Figure 1:**
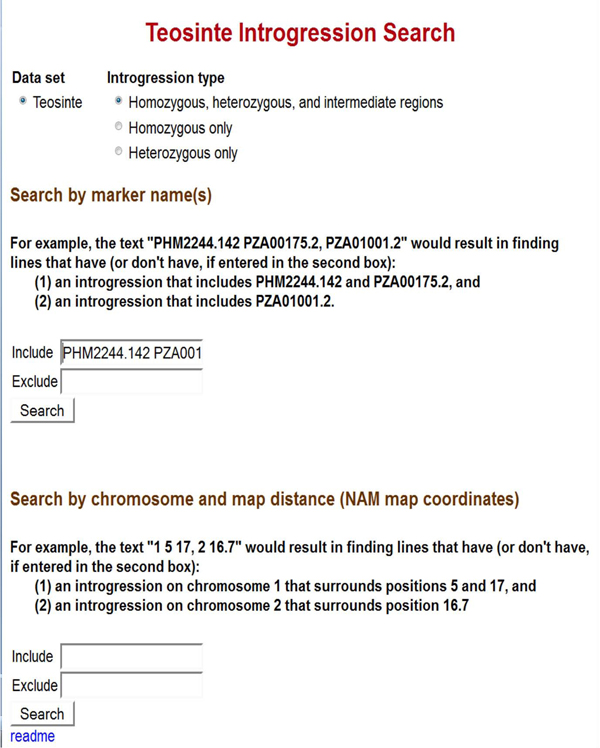
**Initial Search Page.** Queries identify NILs having (or not having) specified donor regions in the genome. Regions can be defined by marker names or by chromosome numbers and map positions corresponding to those of the reference map. This example uses the current maize nested association map[12] as the reference map.

A web browser should now open to the IView search page, similar to Figure 1.

Type " PZA00832.1" in the first "Include" box and press <ENTER>.

**Figure 2:**
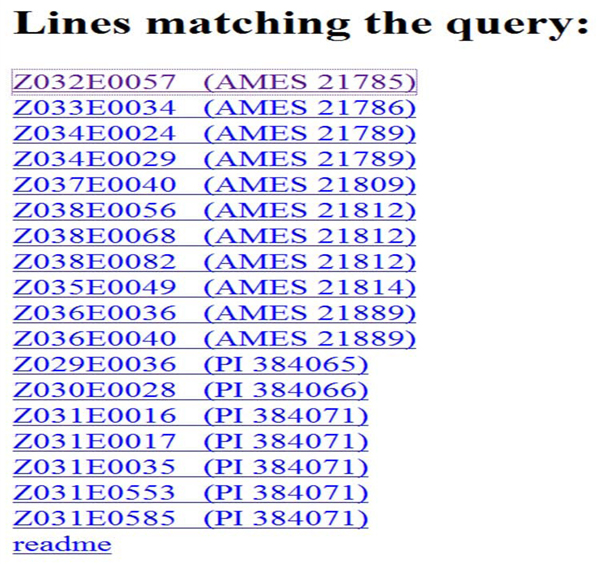
**Query Results.** Links for each of the NILs matching the query result are shown. Each link leads to a NIL Introgression Summary page.

**Figure 3:**
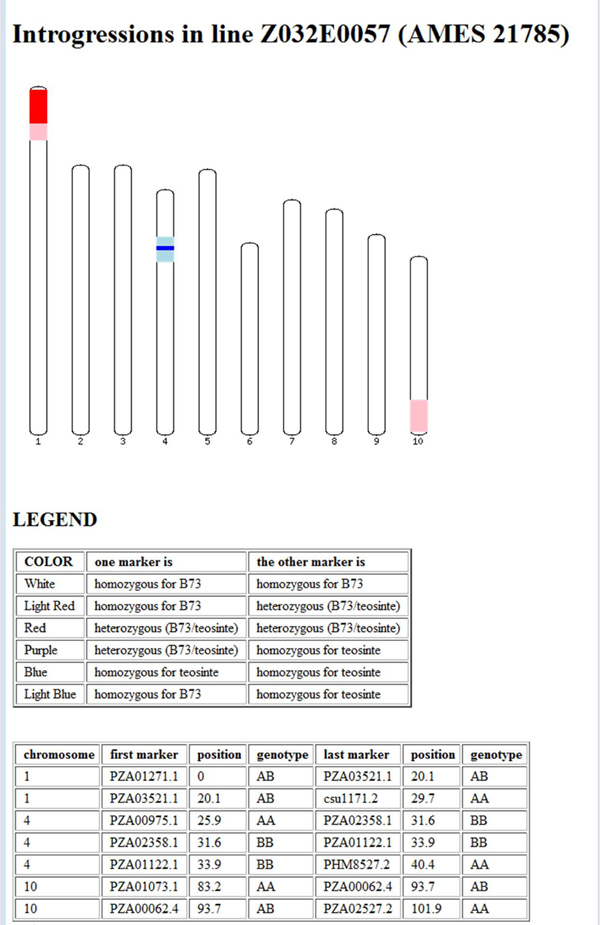
**NIL Introgression Summary.** Introgression information for a NIL is displayed by graphically and in a table.

You should now see a page similar to the one shown in Figure 2. Click on one of the lines listed to see  a NIL Introgression Summary page, similar to the one shown in Figure 3. Scroll down for the legend.

### Input files

 Processing and viewing data with IView requires generating several input files beforehand: a *reference map file, relationships file*, a *raw data file*, and a *configuration file*. We cannot overemphasize the need to format these files correctly. If you encounter any difficulties with preparing these files or running IView, please do not hesitate to contact us. 

#### *reference map file*

A reference genetic map provides a common framework within which to compare single nucleotide polymorphism (SNP) data. Reference map data include the SNP name, the chromosome on which it is located, and the genetic position on the chromosome.  In Table 1 is a representation of the first few lines of a reference map file. This is a tab-delimited file with the following columns: marker name, chromosome number, and marker position. The headers should not be included, but are only included in Table 1 to help clarify what each column represents.

**Table 1:** Representation of a *reference map file*

Marker Name	Chromosome	Marker position
PZA01271.1	1	0
PZA03613.1	1	9
PZA02129.1	1	37
PZA02032.1	1	51

#### *relationships file*

The first line contains headers and the remaining lines contain tab-delimited fields in the following order: sample id, sample name, group name, recurrent parent, donor parent, sample id for a sample that this is a replicate of,  sample id for the F1 of the recurrent and donor parents. If any of the last four fields are not applicable, they can be left blank. However, being blank when they are applicable will result in failure of the program to analyze the data properly. See Table 2 for a representation of this file.

**Table 2:** Representation of a *relationships file*

Sample ID	Sample name	Group	Recurrent Parent	Donor Parent	Replicate of	F1 ancestor
1	B73	Control				
2	B73	Control			1	
3	B73	Control			1	
4	Mo17	Control				
5	Mo17	Control			4	
6	Mo17	Control			4	
7	M0021	IBM	1	4		
8	B73xZ100	F1	1			
9	TIP Z	Inbred	1			8

#### *raw-data file*

This file contains data in a matrix format in which rows represent genotypes at individual markers and columns represent each sample.  The file has up to four parts, the last three of which are relevant to processing data with IView: 

(1) Header section. The beginning of the file can be any text as long as it does not contain the string "[Data]". 

(2) End of Header indicator. The line just before the sample IDs must contain the text "[Data]".

(3) Sample ID line. This line contains a tab character followed by tab-delimited sample IDs. These sample IDs correspond to columns of data in the subsequent lines. Sample IDs in this line must be represented in the *relationships file* in order to be processed.

(4) The remaining lines contain the name of the marker in the first field and data in the remaining fields.

See Table 3 for a representation of this file.

**Table 3:** Representation of a *raw-data file*

[DATA]	sample IDs							
**marker**	**1**	**2**	**3**	**4**	**5**	**6**	**7**	**8**

SNP1	AA	AA	AA	AA	AA	AG	GG	GG
SNP2	AA	AA	AA	AA	AA	AT	AA	AA
SNP3	CC	CC	CT	CC	CC	CC	CC	CC
SNP4	GG	GG	GG	GG	GG	AG	--	--
SNP5	GG	GG	GG	GG	GG	AG	AG	AG
SNP6	AA	AA	--	--	--	AC	CC	CC
SNP7	AA	AA	AA	AA	AA	AG	TT	TT
SNP8	TT	GG	CC	CC	CC	CC	CC	CC

#### *configuration file*

See Table 4 for an example configuration file with explanations. The configuration file is arranged as name/value pairs separated by whitespace (tabs and/or spaces).  

**Table 4:** Sample configuration file.

Key-Value pairs (i.e. file contents)		Explanation
DB_NAME	sample	Name for the new database to be created.
RAW_DATA	sample_data.tab	Data file containing genotype data.
REF_MAP	sample_ref_map.tab	Reference map file.
RELATIONSHIPS	sample_relationships.tab	Relationships file.

## Results and discussion

GBrowse [1] and Ensembl [2] are two of the most popular web-based genome browsers available. After considering these browsers, we decided to create a tool that required less configuration and initial setup for uses specific for displaying introgression lines. Another program related to our problem domain is CSSL Finder, a desktop application for managing introgression data.[3] Unlike IView, it is dependent on Microsoft Excel and is not readily configured for displaying its data on the web.

In comparison with the genome- browsers, IView can easily be installed and used by biologists with minimal computer skills (see Installation section). Our testing volunteer, who has no experience using or installing Perl programs, successfully installed and used IView.

Before being able to display introgression data, introgressions must be determined. Given SNP genotype data for recurrent, donor, and NIL lines, IView can be used to determine the locations of introgressions.

Introgressions can be displayed and queried via IView’s web interface, which comprises three web-pages. First is the initial search page that allows the user to identify NILs that have, or that lack, introgressions in specific locations (Figure 1). On the initial search page, there are two sets of search boxes: one for searching by marker names and the other for searching by chromosome name and reference map position. Next, the Query Results page displays all of the lines matching the query (Figure 2), and allows the user to choose a specific NIL. After choosing a specific NIL, the NIL Introgression Summary page summarizes all of the introgressions in the chosen line, both graphically and in tabular form (Figure 3). 

So far, IView has only been used in-house for displaying and querying Teosinte introgressions in maize NILs. We look forward to seeing it used for introgression lines in others species as well. 

## Conclusions

We have found this tool useful in our own work and expect it to be useful to others working with near-isogenic introgression lines.

## Availability and requirements

Project name: 		IView: Introgression library and visualization tool

Project home page:	http://sourceforge.net/projects/iviewer/

Operating system(s): 	Debian Linux

Programming language: Perl

Other requirements: 

	GD Graphics Library[4]

	MySQL[5]

Major Perl module dependencies (will be automatically installed, if needed) 

CGI::Application[6]

CGI::Application::Plugin::TT[7]

File::Slurp[8]

GD[9]

Template[10]

Plack[11]

License: 	Perl license

## List of abbreviations

CGI: common gateway interface; NIL: near-isogenic line; SNP: single nucleotide polymorphism.

## Competing interests

The author declare that they have no competing interests.

## Authors' contributions

CAB contributed to design, performed programming, and prepared the manuscript. SFG contributed design ideas for the program. MDM contributed design ideas and supervised the project. All authors have read and approved the final version of the manuscript.
